# Long COVID and Physical Therapy: A Systematic Review

**DOI:** 10.3390/diseases11040163

**Published:** 2023-11-09

**Authors:** Juan Carlos Sánchez-García, Andrés Reinoso-Cobo, Beatriz Piqueras-Sola, Jonathan Cortés-Martín, María José Menor-Rodríguez, Raquel Alabau-Dasi, Raquel Rodríguez-Blanque

**Affiliations:** 1 Research Group CTS-1068, Andalusia Research Plan, Junta de Andalucía, 18014 Granada, Spain; jsangar@ugr.es (J.C.S.-G.); bpiquerassola@gmail.com (B.P.-S.); rarobladoc@ugr.es (R.R.-B.); 2 Department of Nursing, Faculty of Health Sciences, University of Granada, 18071 Granada, Spain; 3 Department of Nursing and Podiatry, Faculty of Health Sciences, University of Malaga, Ampliación de Campus de Teatinos, Arquitecto Francisco Peñalosa 3, 29071 Malaga, Spain; andreicob@uma.es (A.R.-C.); rada@uma.es (R.A.-D.); 4 Virgen de las Nieves University Hospital, 18014 Granada, Spain; 5 Área Sanitaria Santiago de Compostela-Barbanza, Subdirección de Humanización y Atención a la Ciudadanía, 15706 Santiago de Compostela, Spain; mariajosemenor@hotmail.com; 6 San Cecilio University Hospital, 18071 Granada, Spain

**Keywords:** long COVID, COVID-19, SARS-CoV-2, physical activity, respiratory exercises, quality of life

## Abstract

Prolonged COVID is a persistent condition following the initial COVID-19 infection, which is characterized by a variety of symptoms that may include fatigue, muscle pain, sleep disturbances, “brain fog”, respiratory, cardiovascular, digestive, neurological and dermatological symptoms. Physical therapy has been identified as a crucial aspect of the management of patients with long COVID, as it can help improve symptoms and overall physical function. The investigation of long COVID poses significant challenges due to the diversity and variability of symptoms, lack of clear diagnostic criteria, and limited understanding of the underlying mechanisms. The aim of this study is to conduct a systematic review of studies conducted in patients with long COVID in conjunction with interventions targeting respiratory function, particularly involving physical activity. To this end, we conducted a systematic review to analyze studies conducted on treatment programs for long COVID based on some form of physical activity. The protocol of the review was registered in the PROSPERO website, and the databases PubMed, Scopus, CINAHL and WOS were searched. Of the 62 initial articles, six were included in the review. The results obtained have positive implications for the advancement of physical activity as a therapeutic intervention for individuals with long COVID-19 and the conceptualization of evidence-based treatment protocols. Statistically significant results have been observed in studies of at least 6 weeks duration, in which inspiratory muscle training exercises are proposed. Further research is needed to better understand long COVID and develop effective treatment strategies.

## 1. Introduction

While many individuals experience an acute phase of the disease, during which they contract and eventually recover from COVID-19, it is crucial to acknowledge that for some patients, the symptoms and health implications can persist over an extended period of time. This prolonged manifestation of symptoms has led to the emergence of a new term in the medical community: “long COVID”.

Long COVID refers to the ongoing and persistent health issues experienced by some individuals who have previously contracted COVID-19 even after the acute phase has resolved. It can involve a wide range of symptoms affecting various organs and systems in the body. As our understanding of this condition continues to grow, it is increasingly clear that COVID-19’s impact extends beyond the initial acute infection and may have long-lasting effects on individuals’ health and well-being [1].

When discussing long COVID, it refers to a syndrome characterized by the continuation of symptoms after the initial COVID-19 infection has resolved. These lingering symptoms can vary in duration, extending anywhere from weeks to even months. Additionally, some individuals may experience the onset of new symptoms after a period of being symptom-free. It is important to note that approximately 20% of individuals who have had COVID-19 will develop symptoms after 5 weeks from the initial infection, while about 10% will experience symptoms reemerging after 12 weeks.

What sets long COVID apart is the fluctuating nature of its symptoms. They may come and go, at times worsening with physical or mental exertion. Importantly, the persistence of these symptoms is not associated with the initial severity of the COVID-19 infection. Both individuals who had mild and severe cases can suffer from long COVID, and the specific symptoms can vary widely [2,3]. 

The symptoms associated with persistent COVID are diverse and may include general symptoms such as fatigue, muscle pain, or sleep disorders as well as specific symptoms affecting the respiratory, cardiovascular, digestive, neurological, and even dermatological systems. One prevalent symptom in long COVID is often referred to as “brain fog”, where individuals may experience difficulties in concentration, memory, and executive function [4,5].

It is worth noting that the population most commonly affected by long COVID tends to be middle-aged women. This demographic prevalence has significant implications, as it not only impacts personal life but also has consequences for the working environment. This can result in economic costs due to limitations in functional capacity, making it a complex and multifaceted health challenge that affects both individuals and society as a whole. As a result, there is a growing need for comprehensive research and medical attention to understand and address the various aspects of long COVID effectively [1].

Physiotherapy is a vital component of the treatment strategy for people with long-term COVID. Physiotherapy plays a multifaceted role in addressing and alleviating symptoms, improving the overall well-being of affected patients.

Fatigue, muscle and joint pain and shortness of breath are common and often debilitating symptoms experienced by people with long-term COVID. Physiotherapists employ a variety of interventions to address these problems. This includes the development of tailored exercise programs aimed at improving not only muscle strength but also flexibility and cardiovascular fitness. These exercises are designed to address the specific needs and limitations of each patient [6,7]

In addition to exercise, physiotherapists may use techniques such as manual therapy. This hands-on approach focuses on improving joint mobility and reducing pain, which can be especially valuable for those experiencing musculoskeletal discomfort associated with long COVID.

Physiotherapists do not limit themselves to treating only physical symptoms. They provide essential education on pacing strategies and energy conservation. These strategies help people to effectively manage their symptoms, avoiding overexertion and optimizing daily activities. In this way, patients can improve their overall physical function and quality of life [8].

In addition, the impact of prolonged COVID extends beyond the physical realm, affecting the psychological well-being of the individual. Physiotherapy also plays a crucial role in addressing these psychological effects. Through guided exercise and other therapeutic interventions, physiotherapists help patients cope with the emotional and psychological challenges associated with prolonged COVID, ultimately contributing to a more holistic and comprehensive approach to care [6,7,8,9].

Long COVID is still an emerging condition, and there is still much that is not known about it. While it is true that this is a well-defined syndrome, long COVID poses a significant challenge for healthcare professionals, as it is a complex and multifaceted condition that can present with a wide range of symptoms. The persistence of symptoms after the initial infection and the lack of clear diagnostic criteria can make it difficult for healthcare professionals to identify and manage long COVID [10]. 

Researching long COVID is a challenging task due to the diversity and variability of symptoms associated with the condition. The wide range of symptoms and their fluctuating nature make it difficult to establish clear diagnostic criteria and to identify a homogenous patient population for study [11,12]. Additionally, the lack of understanding of the underlying mechanisms of long COVID further complicates the research process. This makes it difficult to generate a large enough sample size for studies and to draw clear conclusions about the prevalence and natural history of the condition. Furthermore, the lack of consistent and standardized measurement tools for assessing symptoms and functional impairment in long COVID further exacerbates the difficulties in conducting research in this field. Overall, the variability of associated symptoms and the lack of understanding of the underlying mechanisms of long COVID pose significant challenges to the research of this syndrome, resulting in a relatively scarce amount of data on each of the aspects related to long COVID.

Despite this, many researchers and healthcare professionals are working to understand long COVID, and new studies and research are being conducted to better understand the condition and its impact [12,13,14]. The World Health Organization (WHO) also acknowledged the existence of long COVID and the importance of further research on this topic. 

Long COVID is a multisystemic disorder characterized by the persistence of symptoms beyond the acute phase of SARS-CoV-2 infection. However, the exact pathophysiology and long-term outcomes of this condition remain poorly understood. The variability of symptoms associated with long COVID presents a significant challenge for researchers in terms of identifying consistent patient populations and determining appropriate outcome measures. In particular, the prevalence of respiratory sequelae in long COVID patients highlights the need for further research on the effectiveness of interventions targeting these specific symptoms [10,14,15,16]. 

The aim of this study is to conduct a systematic review of studies conducted in patients with long COVID in conjunction with interventions targeting respiratory function, particularly involving physical activity.

## 2. Materials and Methods

### 2.1. Review Protocol

The methodology employed for the preparation of this report involved a comprehensive systematic review of scientific literature spanning from 2019 to the present day. This review adhered to the established protocol known as Preferred Reporting Items for Systematic Reviews and Meta-Analyses (PRISMA). The PRISMA protocol encompasses a 27-point checklist that systematically covers the most critical components found in an original research article, outlining the process by which these guidelines were meticulously implemented [17]. 

This systematic review was conducted in accordance with a pre-established protocol, which is accessible online at http://www.crd.york.ac.uk/PROSPERO/ (accessed on 3 September 2023) with the registration number CRD42023391811.

### 2.2. Eligibility Criteria 

We selected articles with Randomized Clinical Trial (RCT) methodology, peer-reviewed, in persons of either sex of adult age, that provided information on physical therapies performed after SARS-CoV-2 infection with no restrictions on the language of publication. 

As exclusion criteria, we included studies in which patients used protective masks. We also included studies that were designed to alleviate the sequelae of any other pathology in patients who had been infected with SARS-CoV-2.

### 2.3. Sources of Information

The literature search was performed in the Scopus, PubMed, Cinahl, and WOS databases. A manual search was also performed using reference lists of studies to find other relevant studies. 

The structured language used was obtained using MeSH terms and Health Sciences (DeCS) descriptors. The descriptors used were “long COVID”, “physical therapy” and “post-acute COVID-19 syndrome” using AND, NOT as Boolean operators.

### 2.4. Search Strategy

The following table (Table 1) displays the search strategy employed to conduct this study in each of the consulted databases along with the date of the search and the articles collected in each of them.

### 2.5. Data Extraction Process 

After carrying out the search strategy, the articles found were transferred to the Mendeley web application using the Mendeley web importer tool. They were then organized by folders, according to the database from which they had been obtained, and all duplicates were removed. 

Included studies were RCTs aimed at evaluating physical therapies performed after infection of SARS-CoV-2 patients in adults regardless of sex and published between 2019 and the present. Two reviewers (J.C.-M. and R.R.-B.) independently examined the title, abstract and keywords of each study identified in the search and applied the inclusion and exclusion criteria. The same procedure was applied to potentially eligible full-text articles. Differences between reviewers were resolved by discussion or by a third reviewer (J.C.S.-G.).

Data on quality, patient characteristics, interventions and relevant outcomes were extracted independently by two reviewers (M.I.T.-G. and A.L.-G.).

### 2.6. Data Collection Process and Data Collected

Two reviewers (B.P.-S. and J.C.-M.) independently extracted the following data from each included article: number of participants, participant characteristics, intervention, results, outcomes, conclusions and findings. They also assessed the strengths and weaknesses of each RCT.

We used RobotReviewer software to extract the data and then compared the results with their conclusions. RobotReviewer is a machine learning system designed to support evidence synthesis in the field of evidence-based medicine (EBM). Although it is a highly effective software, it is essential to understand that it does not replace human reviewers in the systematic review process. It was used as a complementary tool to the authors of this review. These reviewers validated and refined the suggestions provided by machine learning as needed.

### 2.7. Methodological Quality Assessment

Methodological quality assessment was performed using the PEDro (Physiotherapy Evidence Database) scale, as the methodology corresponded to a Randomized Clinical Trial (RCT) [18]. The scale comprises 11 items that address aspects such as allocation concealment, blinding, participant follow-up, intention-to-treat analysis, and other criteria related to the rigor of the study design. Each item is scored as 1 if met or 0 if not met, and the total score can range from 0 to 10 with the first item being excluded from the final score. If the clinical trial scores between 9 and 10, it is of very good quality; if it scores between 6 and 8, it is of good quality; if it scores between 4 and 5, it is of fair quality; and if it scores below 4, it is of poor quality. 

### 2.8. Risk of Bias in Individual Studies

The assessment of potential bias within the study was conducted through a two-fold process. Firstly, it was carried out by the machine learning system RobotReviewer [19], which is accessible via the web. This automated system employed algorithms to evaluate the potential biases in the data. Secondly, the authors themselves manually reviewed the data and findings generated by the RobotReviewer system to ensure the accuracy and reliability of the assessments made by the tool. In essence, both automated and human assessments were employed to comprehensively gauge the risk of bias within the study.

### 2.9. Synthesis of Results

Based on the information provided by this review, a series of premises are obtained that will serve to homogenize concepts about the therapies used by health professionals to mitigate or alleviate the effects of SARS-CoV-2 infection in individuals.

## 3. Results

**Figure 1:**
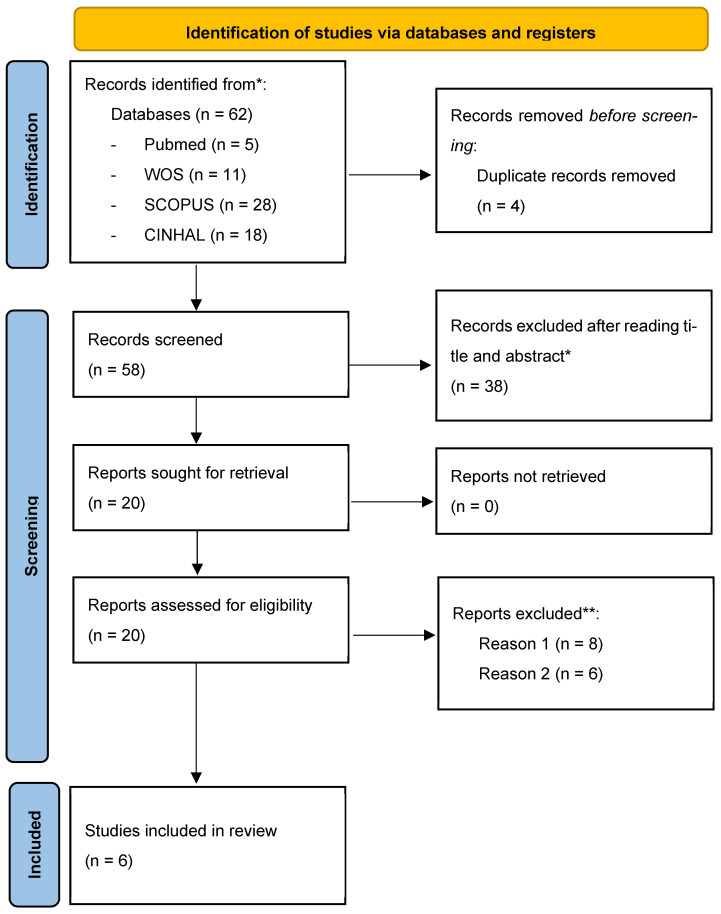
Flow chart (PRISMA guidelines). * They did not meet the inclusion criteria. ** Reason 1: Manuscripts were manuscripts performing physical therapies related to protective masks; Reason 2: Manuscripts did not address post-COVID sequelae but rather sequelae of other pathologies that had been infected by SARS-CoV-2.

After applying the search strategy for articles in the different databases and applying the inclusion and exclusion criteria reported in the methodology, we identified six studies that we included in our review. Figure 1 shows the flow diagram of the articles identified.

Methodological quality was assessed using the PEDro (Physiotherapy Evidence Database) scale, see Table 2, which contains the following criteria: + Yes, - No; P1: The selection criteria were specified (it does not receive a score); P2: Random assignment; P3: Allocation concealment; P4: Similar groups at baseline; P5: Blinding of participants; P6: Blinding of therapists; P7: Blinding of assessor; P8: Drop-outs < 15%; P9: Intention-to-treat analysis; P10: Differences reported in the results between groups; P11: Point estimates and reported variability. 

The least controlled criteria are those related to the following: the concealment of the allocation that guarantees adequate randomization, in addition to the blinding of participants, therapists and evaluators, which affects the results of the study. In relation to drop-outs, the following reasons are reported: personal, difficulty in attending, deterioration. Five of the studies report intention-to-treat analyses (cite 1 to 5), which is an analysis that allows for the control of losses in order to minimize the impact on the results. In the case of the articles chosen for this systematic review, the values range from 5 to 7, thus receiving an average score of 6.17, indicating that the average scientific quality is considered to be of “good quality”.

The risk of bias was assessed using the RevMan program and the RobotReview program, as the latter does not assess selective reporting or other sources of bias but was used to confirm that the analysis performed by the authors coincided with the assessment recorded in RevMan (according to Cochrane criteria). In relation to the analysis, there is no allocation concealment so that selection bias and confounding may occur in the same way as with therapists and raters. In general, studies report a low risk of bias for randomization and selective reporting, while there is a high risk for blinding and incomplete data.

The following table (Table 3) shows the result obtained after performing the risk of bias assessment using the COCHRANE scale.

Table 4 shows a summary of the results of the most outstanding studies in this review. The participants, intervention, results, and conclusions have been selected from the articles, and a column with the statistical significance of the results of each article is highlighted.

## 4. Discussion

The results obtained have positive implications for the promotion of physical activity in patients with long COVID and the design of interventions.

The sample of the Palau et al. study was 26 patients, who performed a 12-week inspiratory muscle training (IMT) home program [15]. The study of Sharma et al. with 30 post-COVID-19 patients does not provide the ages of the patients nor the percentage of women. They carried out a therapeutic treatment protocol 4 days a week for the next 6 weeks, consisting of a pulmonary tele-rehabilitation program based on breathing exercises and therapeutic exercises [24]. Jimeno-Almazán et al. 2023 with 80 non-hospitalized patients with a post-COVID-19 condition were randomized to one of the four programs designed [20]. The programs lasted 8 weeks and consisted of the following: a multicomponent exercise program based on concurrent training of three supervised weekly sessions of aerobic endurance and resistance at low–moderate intensity; an inspiratory muscle training with two standardized daily sessions; a combination of both; and a control group following WHO guidelines for rehabilitation of post-COVID-19 disease [1]. 

Jimeno-Almazán et al. conducted another study in 2022 [21] with 39 participants for 8 weeks (two supervised sessions per week comprising resistance training combined with moderate-intensity aerobic training, plus a third day of continuous supervised light-intensity training). Jimeno-Alamazán’s two studies compared the WHO recommendations with his specifically designed exercise program against long COVID.

Philip et al. conducted an RCT with 192 long COVID patients experiencing dyspnea. They conducted a 6-week online breathing and well-being program, which was developed for people with long COVID experiencing breathlessness [23]. 

The study by Mcnarry et al. conducted an eight-week inspiratory muscle training (IMT) program in 281 adults with long COVID [22].

All studies except McNarry’s had statistically significant results with the intervention group showing statistically significant results compared to the control groups [22]. This may be because the intervention performed by the patients of McNarry et al. was three unsupervised weekly sessions of IMT, on non-consecutive days, for eight weeks [22]. The fact of being unsupervised may have produced such a result. Comparatively, studies such as Palau et al. [15] and Sharma et al. [24] performed the same type of exercises, but the supervision was different from McNarry [22]. The study of Palau et al. [15] was conducted with supervised home visits by a physiotherapist, and in the Sharma et al. study [24], patients performed tele-rehabilitation lung exercises, where the researchers monitored the patients via computer media when they performed physical exercises.

Statistically significant results have been observed in studies of at least 6 weeks duration, where inspiratory muscle training exercises are proposed [25,26]. It is also noted that in the studies proposed by Jimeno-Almazán et al., 2022 [21] and Jimeno-Almazán et al., 2023 [20], which designed an aerobic and resistance exercise program compared to that proposed by the WHO, their results were statistically significant.

The results obtained suggest that physical activity should be incorporated into daily routines rather than being perceived as a separate task to be completed [22,27,28,29,30]. This integration may lead to more sustainable physical activity behavior, as people are more likely to engage in activities that are routine and part of their daily habits and thus improve the quality of life of these patients. In addition, the study highlights the importance of focusing on social and environmental factors, such as social support, as well as individual factors, such as motivation and attitudes, to promote physical activity effectively [14,31,32,33]. These findings provide evidence for the importance of using physical activity in a more holistic way in long COVID patients, as they address individual factors that impact on the social lives of these patients.

However, more studies of this type are needed, as the limitations are clear and may compromise the validity and reliability of the results. These limitations stem from sample sizes, potential for bias, inadequate control of confounding variables and even the cross-sectional approach. It is therefore crucial to take these limitations into account when interpreting and applying the results of such studies in order to ensure accurate interpretation and appropriate use of their results in relation to physical activity and prolonged COVID.

## 5. Conclusions

In conclusion, these investigations support the hypothesis that physical activity interventions may have positive effects on post-COVID recovery. However, to establish the validity of these results, future studies must employ rigorous methods to control for extraneous variables and ensure the generalizability of the findings. Further research is required to establish the robustness of these findings.

## Figures and Tables

**Table 1:** Search strategy used in each of the databases.

Sources	Search Strategy	Filters	Limits	Date of Search	Items
SCOPUS	(TITLE-ABS-KEY-AUTH (long AND covid) AND TITLE-ABS-KEY (physical AND therapy AND modalities) AND NOT TITLE-ABS-KEY (child *)) AND (LIMIT-TO (DOCTYPE, “ar”))	RCT research	2019-	14 January 2023	28
PUBMED	((LONG COVID[Title/Abstract]) AND (“physical therapy modalities” [MeSH Terms])) AND (Therapy/Broad[filter])	RCT Full text	2019-	10 January 2023	5
WOS	long COVID (Topic) and physical therapy (Topic)	Document types: Clinical trial. MeSH Qualifiers: Therapy. MesH Headings: Humans	2019-	8 January 2023	11
CINAHL	long COVID OR post-acute COVID-19 syndrome AND (physical therapy or physiotherapy or rehabilitation)	Limits—Full text; Refereed publications; Human; Age groups: Adult: 19–44 years, Middle Aged: 45–64 years Enlargers—Apply Equivalent Subjects Search Modes—Boolean/Phrase	2019-	15 January 2023	18

**Table 2:** Results of the methodological evaluation according to the PEDro scale.

Reference	P2	P3	P4	P5	P6	P7	P8	P9	P10	P11	Total
Jimeno-Almazán et al., 2023 [20]	+	-	+	-	-	-	+	+	+	+	6/10
Jimeno-Almazán et al., 2022 [21]	+	-	+	-	-	-	+	+	+	+	6/10
Mcnarry et al., 2022 [22]	+	-	-	-	-	-	+	+	+	+	5/10
Palau et al., 2022 [15]	+	-	+	-	-	+	+	+	+	+	7/10
Philip et al., 2022 [23]	+	-	+	+	-	-	+	+	+	+	7/10
Sharma et al., 2022 [24]	+	-	-	+	+	-	+	-	+	+	6/10
Rate of compliance	100%	0%	67%	33%	17%	17%	100%	83%	100%	100%	

**Table 3:** Risk of bias assessment.

Trial	Design	Random Sequence Generation	Allocation Concealment	Blinding of Participants and Personnel	Blinding of Outcome Assessment
Palau et al., 2022 [15]	RCT	+	+	?	+
Sharma et al., 2022 [24]	RCT	?	?	?	+
Jimeno-Almazán et al., 2023 [20]	RCT	+	?	?	?
Jimeno-Almazán et al., 2022 [21]	RCT	+	?	?	?
Philip et al., 2022 [23]	RCT	+	+	?	+
Mcnarry et al., 2022 [22]	RCT	+	+	?	?

**Table 4:** Results included in the systematic review.

Author	Participants	Interventions	Outcomes	Conclusion	Finding
Palau et al., 2022 [15]	26 post-discharged patients with long COVID	They assessed a 12-week home-based IMT program in 26 post-discharge COVID-19 patients	At 12 weeks, the mean pp-peakVO_2_ was higher in the IMT group compared to controls	IMT significantly increased pp-peakVO_2_ in post-COVID patients versus controls	↑ sig increase
Sharma et al., 2022 [24]	30 post-COVID patients with respiratory complications and chronic COVID	They evaluated a pulmonary tele-rehabilitation program including breathing exercises and therapeutic exercises in 30 post-COVID patients with respiratory complications and chronic COVID compared to conventional care	Patients receiving tele-rehabilitation showed significant improvement in dyspnea, fatigue, and ability to rehabilitate at home versus conventional care	Pulmonary tele-rehabilitation was a valuable service for post-COVID patients with respiratory complications	↑ sig increase
Jimeno-Almazán et al., 2023 [20]	80 non-hospitalized adults with post-COVID-19 conditions	They compared two 8-week exercise programs in 80 non-hospitalized adults with post-COVID conditions: (1) multicomponent exercise with concurrent resistance and endurance training versus (2) a program combining inspiratory muscle training, physical exercise and self-management	The multicomponent exercise program elicited significant improvement in fatigue, depression, fitness, quality of life, symptoms, dyspnea, strength, and severity versus the other program	Multicomponent exercise with concurrent training showed more effectiveness than the other program in post-COVID conditions	↑ sig increase
Jimeno-Almazán et al., 2022 [21]	39 patients	They compared 10 weeks of tailored supervised therapeutic exercise versus self-management recommendations in the WHO rehabilitation leaflet in 39 patients with post-COVID conditions	Supervised exercise showed significantly better cardiovascular fitness, strength, quality of life, fatigue, depression, and functional status than self-management controls	Supervised therapeutic exercise was a more effective intervention than self-management in post-COVID conditions	↑ sig increase
Philip et al., 2022 [23]	192 people with long COVID	They assessed a 6-week online breathing and well-being program developed for 192 people with long COVID experiencing breathlessness	The online breathing program improved the mental component of HRQoL and breathlessness VAS while running versus controls	The online breathing program improved mental HRQoL and breathlessness in people with persisting post-COVID symptoms	↑ sig increase
Mcnarry et al., 2022 [22]	281 adults recovering from self-reported COVID-19	They compared IMT versus control in 281 adults recovering from self-reported COVID-19	No difference between groups in KBILD total score but clinically meaningful IMT improvements in breathlessness, chest symptoms, and respiratory muscle strength	IMT may represent an important home-based post-COVID rehabilitation strategy, improving breathlessness, chest symptoms, respiratory muscle strength and estimated aerobic fitness	↓ sig decrease

↑—Means increase; ↓—Means decreaseIMT—inspiratory muscle training; pp-peakVO2—post-COVID peak oxygen uptake; KBILD—King’s Brief Interstitial Lung Disease; HRQoL—health-related quality of life; VAS—visual analogue scale.

## Data Availability

Data are available on request from the corresponding author.
